# Assessment of Preptin peptide level in the sera of rachitic children and in breast milk of their mothers

**DOI:** 10.1186/s13052-019-0628-8

**Published:** 2019-03-07

**Authors:** Gihan M. Bebars, Salem A. Sallam, Shereen S. Gaber, Alshimaa H. Abdelaziz

**Affiliations:** 1 Faculty of Medicine, Pediatric Department, Minia University, Minia, Egypt; 2 Faculty of Medicine, Biochemistry Department, Minia University, Minia, Egypt; 3 Minia General hospital, Minia, Egypt

**Keywords:** Preptin, Breastmilk, Rickets, Peptide hormone

## Abstract

**Background:**

Preptin is a 34-residue pancreatic hormone that stimulates osteoblast proliferation and reduces osteoblast apoptosis.

**Research aims:**

To measure levels of serum Preptin in rachitic children and in breastmilk of their mothers and to compare with levels in healthy non-rachitic children.

**Methods:**

Thirty children with rickets and another 30 non-rachitic age and sex matched controls were subjected to detailed history, physical examination including anthropometric measurements, assessment of signs of rickets and laboratory measurement of serum vitamin D, calcium, phosphorus, alkaline phosphatase and Preptin. Mothers’ breast milk Preptin were also measured.

**Results:**

Significantly lower serum Preptin (*p* < 0.001) in rachitic children with a significant negative correlation between serum Preptin and alkaline phosphatase (*P* < 0.0001). Lower breastmilk Preptin levels in mothers of rachitic children (*P* < 0.001) with a negative correlation between breastmilk Preptin and both maternal weight and BMI(*P* < 0.01&*P* < 0.02). Mothers’ milk Preptin is positively correlated with serum Preptin and calcium in non-rachitic children(P < 0.001&0.04), but negatively correlated with their mothers’ age (*P* < 0.01).

**Conclusion:**

Preptin may play a role in the etiology of rickets in children. Further studies are recommended to evaluate Preptin role in treatment of rickets in children.

## Background

Rickets is a disease of growing children characterized by a failure or delay in endochondral calcification of the growth plates of long bones [1] accompanied by osteomalecia [2] that may be caused by either vitamin D deficiency and decreased calcium intake or high phytate intake. Africa, Middle East and Asia were reported to have the highest prevalence of rickets world-wide [2].

Preptin is a 34-residue pancreatic hormone synthesized primarily in the pancreas, salivary gland, mammary tissue, and kidneys [3]. It is shown to be anabolic to bone in vitro and in vivo and its bone activity resides within the [1–16] N-terminal fragment of Preptin peptide. The truncated fragment of Preptin is enzymatically unstable but, it provides an attractive framework for the creation of stable analogues using various peptidomimetic techniques [4]. *Cornish* et al in their study [5] reported that Preptin [1–16] retained the full Preptin-like bone-anabolic activity in vitro using fetal cultures of primary rat osteoblasts at 10^− 10^ and 10^− 11^ M. Furthermore, the truncated fragment increased the bone area and percentage of mineralizing bone surface in mature male mice, indicating that the smaller peptide fragment also enhances bone growth in vivo [5].

Preptin stimulates osteoblast proliferation and reduces osteoblast apoptosis through the MAP-kinase pathway, and this leads to an increase in bone area and mineralization [5]. Previous studies reported low bone mineral densities to be associated with lower serum Preptin levels [5, 6].

This study aimed to measure serum Preptin level in rachitic children and breastmilk Preptin of their mothers, also, to compare with their levels in non-rachitic children and their mothers.

## Patients and methods

### Study setting and population

A case control study initiated after obtaining the study approval from the local ethical committee of the Minia Children and Maternity University hospital (code: 45 C at 2/12/2014). The study was conducted according to the declaration of Helsinki. Parents and/caregivers approved to participate in our study and this was after explaining the purpose of the study to them during counseling and after receiving ethics committee approval.

Studied children were recruited over the period from January 2015 to October 2015 from Children and Maternity Minia University Hospital which is considered as the only referral and teaching hospital in Minia governorate, Egypt. This hospital provides a wide range of health care services and serves urban and rural populations from near and far districts in Minia Governorate.

#### Sample size calculation

Sample size calculated by using software G* power 3.1.9.2. A priori power analysis to calculate sample size for Means: difference between two independent means. The required sample size was estimated based on the following conditions: α error probability = 0.05, Power (1- β error probability) = 0.85 and Cohen’s effect size (d = 0.8) [7]. Accordingly, the sample size was estimated. The final number was 30 participants in each group.

All studied children with age range from 6 months up to 2 years old of both sexes were on exclusive breastfeeding for 6 months of age then complementary feeding started with homemade foods, fortified cereals and routine vitamin D supplementation 400 IU/day.

Studied children were subjected to detailed medical history, physical examination including; anthropometric measurements (weight, length and head circumference) and evaluated for any signs of rickets including;(macrocephaly, rosary beads, marfan sign, delayed teething, bone deformities). Laboratory assessment of serum calcium, phosphorus, alkaline phosphatase, 25 hydroxy vitamin D, serum Preptin and mothers’ breast milk Preptin levels were done in biochemistry department of the Faculty of Medicine. Lastly X ray over wrist joint to confirm diagnosis of rickets (expansion of the metaphysis, irregularity of the epiphyseal margin, a brush−like appearance, cupping and general osteopenia) determined by a radiologist at our institution. Studied children were classified according to previous clinical and laboratory investigations into 2 groups:

Group A: 30 children with vitamin D deficiency rickets.

Group B: 30 children with no clinical or laboratory evidence of rickets and normal vitamin D levels.

#### Exclusion criteria

1. Patients with skeletal problems as (skeletal dysplasia).
2. Patients with metabolic bone diseases including hypophosphatasia.
3. Patients with hypophosphatemic vitamin D–resistant rickets.
4. Vitamin D–deficiency rickets associated with an underlying disease such as fat malabsorption, liver disease, renal insufficiency and illnesses necessitating total parenteral nutrition.

#### Blood and milk sampling and processing

From all studied children 3 ml venous blood samples were collected, centrifuged at 3000 rpm for 15 min to separate serum and divided into two tubes: the first tube to assess serum calcium, phosphorus, alkaline phosphatase and 25 OH vitamin D level. The second tube stored at-20 °C to measure serum Preptin level by Eliza. From mothers 3 ml breast milk samples were collected and stored at − 20 °C to measure Preptin level in breast milk. Breast milk samples were separated by centrifuging twice at 3000 rpm for 10 min. After the first centrifugation, the thick fat layer at the top of the tube was discarded with a sterile toothpick, then the aqueous fraction was collected, centrifuged, filtered then was assayed immediately.

#### Measurement

- Measurements of serum and mother’s milk Preptin levels by Eliza. (Catalog No: MBS2514802, WKEA MED. Changchun. China), detection range of the kit: 12-480 pg /ml. Reported Preptin level concentration in adult human milk ranges from 9.72 ± 2.26 ng/mL to14.32 ± 3.06 ng/mL [3]*.*
- *Total calcium* was measured using calcium *v*/v kit. SPINREACT S.A.U./ SPINREACT, S. A. Ctra. Santander Coloma ESPANA, according to the manufacturer protocol.
- *Alkaline phosphatase* using the alkaline phosphatase liquicolor (DEA Buffer, DGKC) kit, Human Gesellschaft Germany.
- *Phosphorus* using phosphorus liquirapid kit, Human Gesellschaft Germany.

According to laboratory levels, normal value of calcium is (8. 5-11 mg/dl), normal value of phosphorus is (2.4–4 .1mg/dl) and normal value of alkaline phosphatase is (3 5-135 IU/L).

#### Determination of serum 25(OH) vitamin D levels [vitamin D]

Serum levels of 25(OH) vitamin D were determined using automated chemi - luminescent microparticle immunoassay commercially available (Architect, Abbott diagnostics, USA).

25(OH) Vitamin D levels were categorized as deficient (< 20 ng/mL), insufficient (≥20 and < 30 ng/mL), or sufficient (≥30 ng/mL) value.

#### Statistical methods

Data were entered and analyzed by SPSS version 19. Graphics were done by Excel. Quantitative data were presented as mean and standard deviation while qualitative data presented as frequency distribution. Comparison between groups was done by Man-Whitney test and chi-square test. Spearman correlation was used. Probability of less than 0.05 was used as cut off for significance.

## Results

Rachitic children had significantly lower mean body weight, length values (*P* < 0.04& 0.002) and significantly larger head circumference (< 0.005) than non-rachitic children. (Table 1).

**Table 1:** Comparison between rachitic children and their mothers with healthy controls and their mothers regarding their demographic data and anthropometric measurements

Value	Rachitic children group *n* = 30	Non-rachitic children group *n* = 30	*P* value
Age (Month)			
Range	8–24	9–24	0.9
Mean ± SD	15.3 ± 5.1	15.2 ± 4.6	
Sex: n (%)			
Male	17(56.7%)	18(60%)	0.8
Female	13(43.3%)	12(40%)	
Weight (Kg)			
Range	7-13	8–12	0.04*
Mean ± SD	9.3 ± 1.7	10.4 ± 1.4	
Length (Cm)			
Range	67–90	70–93	0.002**
Mean ± SD	75.01 ± 6.5	82.2 ± 7.4	
HC (Cm)			
Range	45–54.5	45–51	0.005**
Mean ± SD	50.2 ± 2.7	47.9 ± 1.5	
Maternal age(year)			
Range	19-39	20-32	0.001**
Mean ± SD	30.8 ± 5.1	25.1 ± 3.9	
Maternal BMI(kg/m2)			
Range	20-30	20-27	0.03*
Mean ± SD	24.7 ± 3.3	22.7 ± 2.1	
Parity (n)			
Range	1-7	1-4	0.08
Mean ± SD	3.2 ± 1.6	2.2 ± 1.2	

Quantitative data compared by Mann-Whitney test, qualitative data by chi square test

significant (*P* < 0.05 *)

Highly significant (*P* < 0.01 **)

HC (head circumference)

Mothers of rachitic children group were older in age with higher BMI values compared to mothers of non-rachitic children (*P* < 0.001&0.03 respectively) but no difference regarding parity(Table 1).

Rachitic children have; rachitic rosary (66.67%), macrocephaly (40%), delayed teething (33.3%) and enlargement of the wrists (83.3%). (data not shown in tables).

Rachitic children have normal calcium level but lower phosphorus and vitamin D levels with higher alkaline phosphatase in comparison to control (*P* < 0.001). (Table 2).

**Table 2:** Comparison between rachitic children and controls regarding some laboratory data

Value	Rachitic children *n* = 30	Healthy children *n* = 30	*P* value
Total Calcium (mg/dl)			
Range	7. 5-10.9	8. 7-10.7	0.4
Mean ± SD	9.4 ± 0.8	9.6 ± 0.6	
Phosphorus(mg/dl)			
Range	1. 1-2.3	2. 5-4.1	0.001**
Mean ± SD	1.6 ± 0.3	3.3 ± 0.4	
Alkaline phosphates (IU/L)			
Range	80–374	45–90	0.001**
Mean ± SD	229.4 ± 84.6	71.4 ± 13.9	
Serum 25(OH) Vit. D (ng/dl)			
Range	7-12	22–30	0.001**
Mean ± SD	7.69 ± 2.34	23.25 ± 2.05	
Serum Preptin (ng/l)			
Range	3. 2-9.3	4. 6-12.5	0.001**
Mean ± SD	6.3 ± 1.5	8.3 ± 1.8	
Breast milk Preptin (ng/l)			
Range	3. 8-9	4. 8-13.8	0.001**
Mean ± SD	6.01 ± 1.6	8.4 ± 3.3	

** means statistically significant

**Fig. 1:**
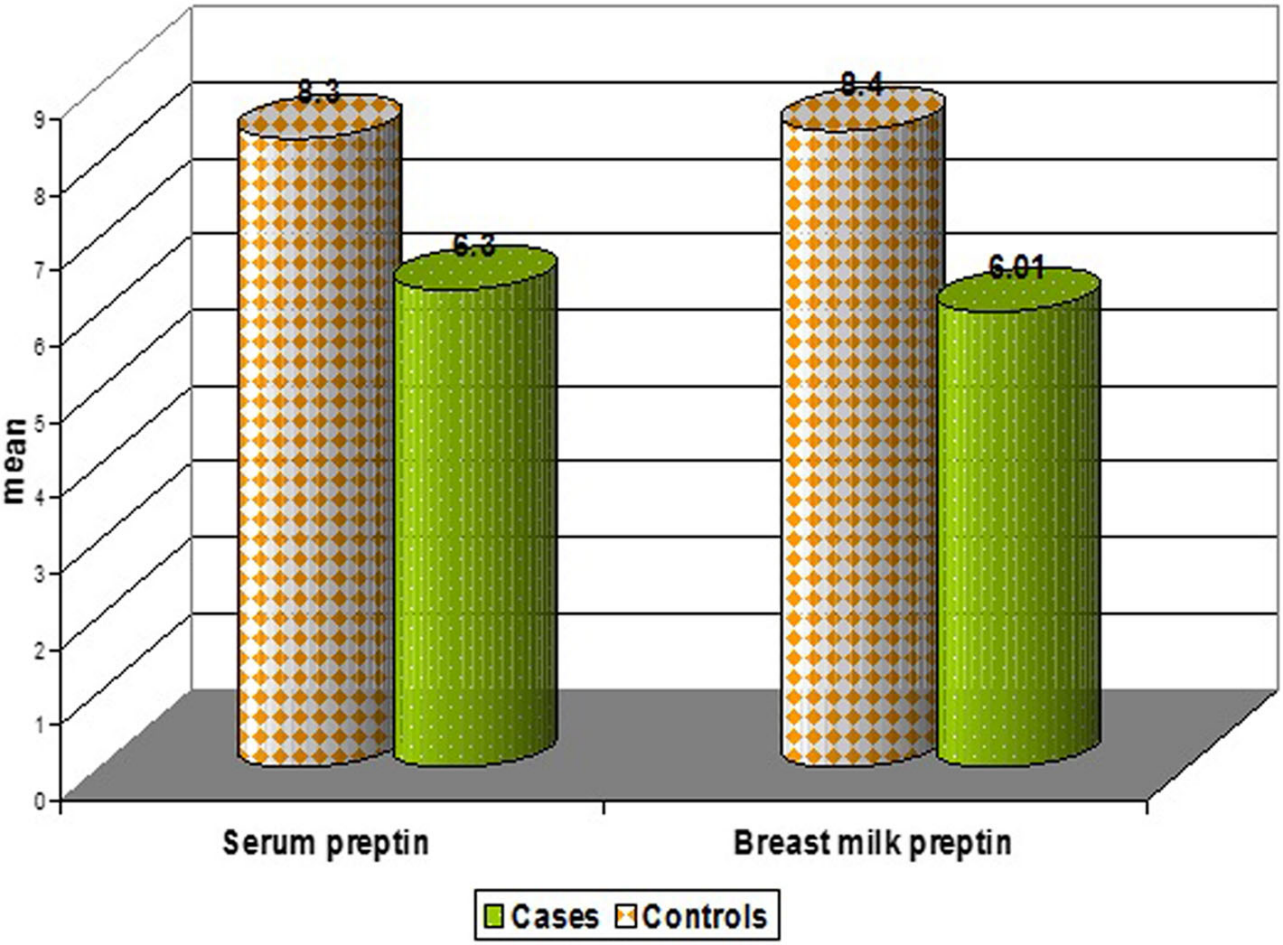
Correlation between serum Preptin (ng/l) and alkaline phosphatase(IU/L) in rachitic children

Lowered serum and breastmilk Preptin levels in rachitic children in comparison to control group (P < 0.001) (Table 2). Serum Preptin was negatively correlated with serum alkaline phosphatase in rachitic children (*p* = 0.001&r = − 0.97) (Fig. 1 & Table 3). A positive relation was found between serum Preptin and serum calcium in control group (*P* = 0.04&r = 0.53) (Table 4). 25(OH) Vitamin D level in rachitic children was positively correlated with their breast milk Preptin (*P* = 0.019&r = 0.42). (Table 3).

**Table 3:** Correlation between serum Preptin(ng/l) in rachitic children and breast milk Preptin(ng/l) with different variables

variables		Serum Preptin (ng/l)		Breast milk Preptin (ng/l)	
		r	P	r	P
Serum Preptin (ng/l)				- 0.04	0.7
Demographic data of Infants	Age (mo)	0.30	0.1	- 0.09	0.6
	Weight(kg)	0.22	0.2	- 0.22	0.2
	Length(cm)	0.27	0.2	- 0.19	0.2
	HC(cm)	0.32	0.08	- 0.29	0.1
Demographic data of mother	Age (year)	0.21	0.2	- 0.06	0.7
	Parity(n)	0.12	0.5	- 0.02	0.8
	Weight(kg)	0.04	0.8	- 0.45	0.01*
	Height(cm)	0.10	0.5	- 0.09	0.6
	BMI(kg/m2)	0.02	0.8	−0.42	0.02*
Laboratory data	Calcium(mg/dl)	0.24	0.1	0.26	0.1
	Phosphorus (mg/dl)	0.03	0.8	0.03	0.8
	Alkaline phosphatase (IU/L)	−0.97	0.001**	- 0.14	0.4
	Serum 25(OH) Vitamin D(ng/dl)	0.190	0.314	0.425	0.019**

Pearson and Spearman correlation

*significant (*P* < 0.05) **highly significant (*P* < 0.01)

**Table 4:** Correlation between serum Preptin (ng/l) of controls and breast milk Preptin (ng/l) with different variables

variable		Serum Preptin (ng/l)		Breast milk Preptin (ng/l)	
		r	P	r	P
Serum Preptin (ng/l)				0.87	0.001**
Demographic data of Infants	Age (mo)	- 0.35	0.1	0.14	0.6
	Weight(kg)	0.37-	0.1	0.20	0.2
	Length(cm)	- 0.42	0.1	0.31	0.2
	HC(cm)	- 0.16	0.2	0.05	0.8
Demographic data Of mother	Age (year)	- 0.03	0.9	−0.59	0.01*
	Parity (no)	- 0.15	0.7	−0.50	0.05
	Height(cm)	0.01	0.1	−0.24	0.3
	Weight(kg)	- 0.19	0.4	0.16	0.5
	BMI(kg/m2)	0.07	0.8	−0.24	0.3
Laboratory data	Calcium (mg/dl)	0.53	0.04*	−0.10	0.7
	Phosphorus (mg/dl)	0.001	0.9	0.05	0.8
	Alkaline phosphatase (IU/L)	0.008	0.9	−0.22	0.4
	Serum 25(OH) Vitamin D (ng/dl)	0.058	0.838	0.496	0.060*

Pearson and Spearman correlation

*Significant (*P* < 0.05) **Highly significant (*P* < 0.01)

**Fig. 2:**
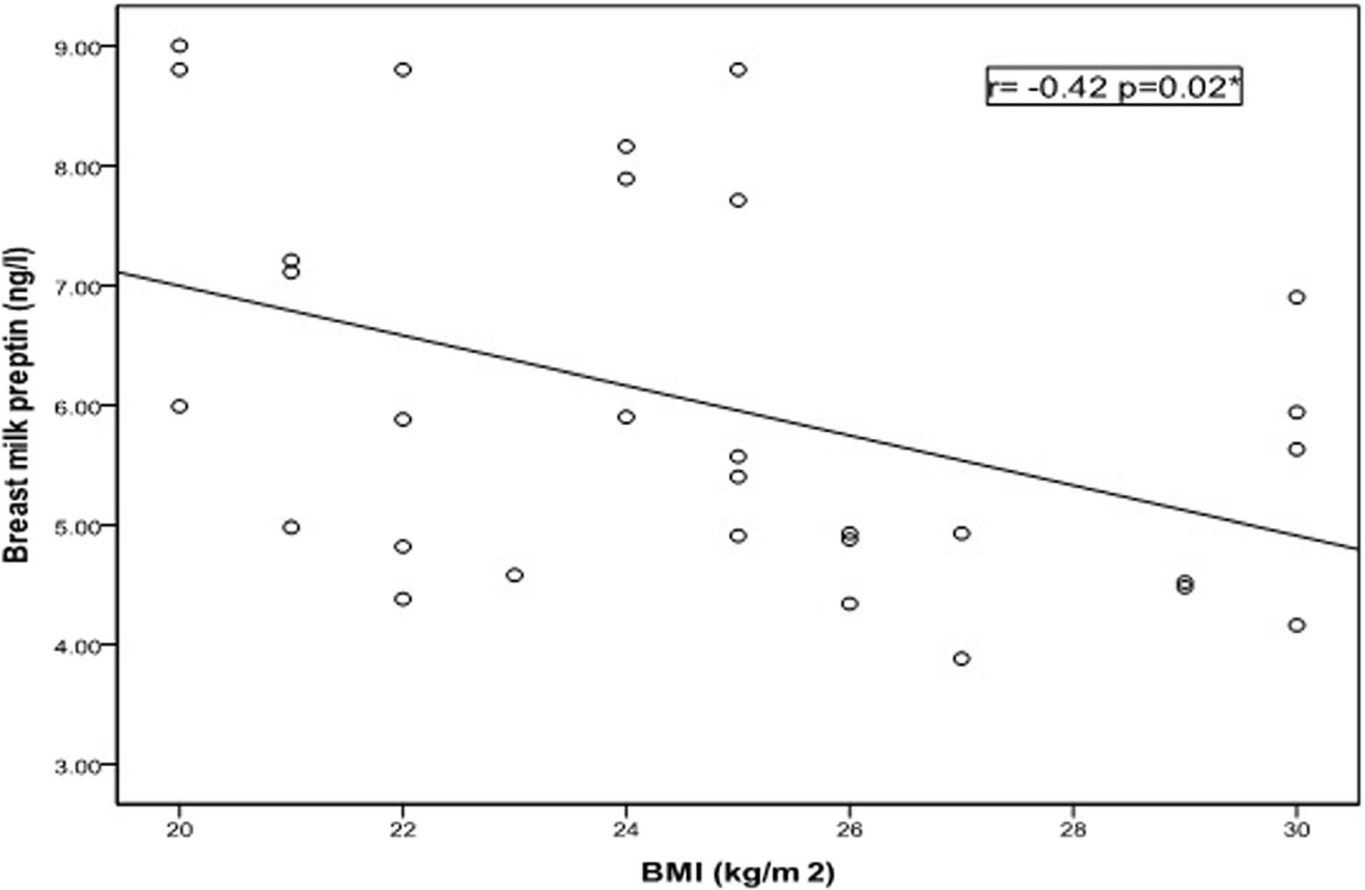
Correlation between breast milk Preptin (ng/l) and mothers’ BMI (kg/m2) in rachitic children group

Breastmilk Preptin was negatively correlated with maternal body weight (P = 0.01 & r = − 0.45) and BMI (*P* = 0.02& r = − 0.42) (Fig. 2) in rachitic children group. In control group mother’s milk Preptin was positively correlated with serum Preptin of their children, but negatively correlated with maternal age (*P* = 0.001&r = 0.87) (P = 0.01&r = − 0.59) respectively.

## Discussion

Gender and age were not related to development of rickets in studied children. This finding agreed with *Tom* et al.*,* and *Yasar Sen* et al., who reported no relation between rachitic infants and age or sex [8, 9]. Rachitic children in our study found to have lower length values and lower mean body weight than non-rachitic children and this agrees with the study of Graff et al. [11], and *Rooze* et al. [12], *but,*this was not the same for *Yasar Sen* et al.*,* as the difference between rachitic children and controls regarding their weight and height was insignificant [9].

BMI in mothers of rachitic children were higher than mothers of control group which may be a risk factor for vitamin D deficiency. Studies reported that pregravid obese women had lower mean serum 25(OH) D concentrations and a higher prevalence of vitamin D deficiency that may lead to decrease stores in newborn and breast milk and give risk of infants’ vitamin D deficiency [13].

The estimated difference in serum calcium level between rachitic children and controls was insignificant that may be related to compensatory increase in PTH. This finding comes in accordance with *Baroncelli* et al. [14] On the reverse Graff et al.*,* reported significant decrease in serum calcium level in rachitic children in relation to controls [11].

Significantly lowered phosphorus levels in rachitic infants than controls were documented in the current study and this agree with *Tiosano and Hochberg* study as they stated that hypophosphatemia is a common denominator in rickets, that prevents apoptosis in the hypertrophic cells in the growth plate. In the absence of apoptosis, the hypertrophic cells accumulate in the growth plate and form the rachitic bone [15].

A higher serum alkaline phosphatase level in rachitic children than healthy controls were encountered in this study. Raised serum levels of alkaline phosphatase indicates a state of increased bone turnover so, it is used as a bone formation marker that correlates positively with the severity of rickets and Osteomalecia [16].

Preptin stimulates osteoblast proliferation and reduces osteoblast apoptosis so, Preptin is involved in bone anabolism and was found to contribute to the bone mass preservation observed in hyperinsulinaemic states [5, 6].

We found that mean serum Preptin in rachitic children was significantly lower than in healthy controls and this finding was close to the study of *Ning,* et al.*,* and *Ozkan* et al.*,* who documented lower levels of Preptin in patients with osteopenia and osteoporosis, with a positive correlation between bone mineral density(BMD) and Preptin levels [5, 17]. To the best of our knowledge the relation between serum Preptin level and rickets in children hasn’t been studied before.

In rachitic children a strong negative correlation was found between serum Preptin and serum alkaline phosphatase levels and this agree with *Ning,* et al.*,* who found an inverse relation between serum Preptin level and serum alkaline phosphatase level in patient with low BMD [5]. As alkaline phosphatase is positively correlated with the severity of the disease [16] so, we can also suggest that serum Preptin can be used as a marker for severity of rickets in children.

The present study detected lowered maternal milk Preptin levels in rachitic group than in control group indicating that lowered breastmilk Preptin may have a role in the development of rickets in children. To the best of our knowledge no studies have evaluated serum or breast milk Preptin in rachitic children or their mothers to establish a clear relationship between them.

In addition, a positive correlation was found between 25 OH vitamin D level in rachitic children and their mothers’ breastmilk Preptin suggesting an effect of breast milk Preptin on vitamin D status of children.

In this study it was observed that mother’s milk Preptin levels decrease with advance of age. Indeed, this observation needs further studies to document any change in breastmilk Preptin in relation to mothers’ age.

Moreover, a significant negative correlation between mother’s milk Preptin concentration and both maternal weight and BMI in rachitic group were documented. Previous studies investigated Preptin changes in relation to BMI index as Ozkan et al. [17], who found a positive correlation between BMI and serum Preptin and suggested that Preptin is involved in the etiopathology of different diseases from obesity and PCOS to diabetes, osteoporosis and osteopenia.

The importance of nutritional hormones to maintain skeletal health has been previously documented [18]. But up till now no data regarding breastmilk Preptin in obese mothers are available for comparison with our data and the available data are about the serum concentration of Preptin only and showed an elevated level of Preptin in relation to BMI [17].

Recent studies were performed to detect the role of peptide hormone Preptin, as a novel bone-anabolic agent for the treatment of osteoporosis ( [19] and another one studied the effect of In vivo administration of Preptin and reported an increased bone area and mineralizing surface in adult mice. In addition, they provide additional evidence for anabolic skeletal effects of peptides derived from pro-IGF-II [20]. From all previous studies and our findings regarding Preptin concentration in serum of rachitic children and in breastmilk of their mothers we would suggest a role of Preptin in pathogenesis of rickets.

## Conclusion

Serum Preptin and breast milk Preptin were found to be lower in rachitic children and their mothers respectively in comparison to non -rachitic. Serum Preptin is inversely correlated with serum alkaline phosphatase and breastmilk Preptin is positively correlated with 25 OH vitamin D level in rachitic children, suggesting a role of Preptin in rickets and may indicate severity. Further studies are needed to search for the role of Preptin in bone metabolism and the possibility of its use in treatment of rickets and other diseases with osteoporosis. Also, Further studies are recommended on a larger scale to study the effect of maternal supplementation with vitamin D on their breast milk Preptin concentrations.

## Abbreviations

BMD: Bone mineral density
BMI: Body mass index
